# Perioperative Systemic Inflammation in Lung Cancer Surgery

**DOI:** 10.3389/fsurg.2022.883322

**Published:** 2022-05-20

**Authors:** József Furák, Tibor Németh, Judit Lantos, Csongor Fabó, Tibor Géczi, Noémi Zombori-Tóth, Dóra Paróczai, Zalán Szántó, Zsolt Szabó

**Affiliations:** ^1^ Department of Surgery, Faculty of Medicine, University of Szeged, Szeged, Hungary; ^2^ Department of Neurology, Bács-Kiskun County Hospital, Kecskemét, Hungary; ^3^ Department of Anesthesiology, Faculty of Medicine, University of Szeged, Szeged, Hungary; ^4^ Department of Pathology, Faculty of Medicine, University of Szeged, Szeged, Hungary; ^5^ Department of Medical Microbiology, Faculty of Medicine, University of Szeged, Szeged, Hungary; ^6^ Department of Thoracic Surgery. Medical School, University of Pécs, Pécs, Hungary; ^7^ Institute of Surgical Research, Faculty of Medicine, University of Szeged, Szeged, Hungary

**Keywords:** thoracotomy, video-assisted thoracic surgery, non-intubated, one-lung ventilation, systemic inflammation, immune cells, cytokines

## Abstract

Systemic inflammation (SI) is a response of the immune system to infectious or non-infectious injuries that defends the body homeostasis. Every surgical intervention triggers SI, the level of which depends on the extent of damage caused by the surgery. During the first few hours after the damage, the innate or natural immunity, involving neutrophils, macrophages, and natural killer cells, plays a main role in the defense mechanism, but thereafter the adaptive immune response ensues. The number of leukocytes is elevated, the levels of lymphocytes and natural killer cells are reduced, and the cytokines released after surgery correlate with surgical damage. Minimally invasive thoracic surgery procedures induce less inflammatory response and reduce the immune defense in patients to a more moderate level compared with the open surgery procedures; this immunosuppression can be further diminished in spontaneous ventilation cases. The normal functioning of the immune defense is important in controlling the perioperative circulatory tumor cells. Moreover, elevated levels of inflammatory cytokines before immune therapy have a negative impact on the response, and significantly shorten the progression-free survival. Clinically, the lower are the levels of cytokines released during lung surgery, the lesser is the postoperative morbidity, especially pneumonia and wound infection. The return to normal levels of lymphocytes and cytokines occurs faster after spontaneous ventilation surgery. The use of locoregional anesthesia can also reduce SI. Herein, we review the current knowledge on the effects of different operative factors on postoperative SI and defense mechanism in lung cancer surgery.

## Introduction

Systemic inflammation (SI) is an immune response to infectious or non-infectious injury in the body, aimed at restoring the normal anatomy and function of the damaged organ and eradicating infectious agents. Although the initial step in this process is localized inflammation with local symptoms, this response can develop into a systematic reaction or overreaction, termed as systemic inflammatory response syndrome (SIRS). Although SIRS can be described as sepsis in the case of infection, it can be a non-infectious process as well, for example, in surgical stress cases (1, 2). The terminology and diagnostic criteria of the SI have changed with time (3). The diagnosis of SIRS is based on clinical signs (body temperature, heart rate, respiration rate, and level of white blood cells) and the presence of proinflammatory cytokines and mediators in the serum (1). For precise diagnosis and prognosis of the SI, currently, the sepsis-3 definition is widely used focusing on the organ functions, and the diagnosis is based on the sequential (or sepsis-related) organ failure assessment (SOFA) score (respiration-arterial oxygen pressure, coagulation-platelets level, liver-bilirubin level, cardiovascular-hypotension, central nervous system-Glasgow Coma Score, renal-creatinine) (4, 5).

With regard to the pathophysiological background of SI, in the first few hours after the damage, innate or natural immunity, involving neutrophils, macrophages, natural killer cells, plays a major role in the early defense mechanism, but it is soon followed by the adaptive immune response . In both types of adaptive immune responses (i.e., humoral and cell-mediated), lymphocytes (T-helper cells, cytotoxic T cells, regulatory T cells, and B cells) play a major role together with cytokines. The two types of immune responses interact very closely with each other.

Surgical trauma, including thoracic surgical procedures, induces SI through systemic proinflammatory and compensatory anti-inflammatory responses (6, 7). In lung cancer surgery, there are two important aspects of SI, viz., postoperative morbidity and control of tumor cell spreading (1, 6, 7). The currently used approaches in lung cancer surgery are open thoracotomy and minimally invasive thoracic surgery (MITS) procedures, such as video-assisted thoracic surgery (VATS) and robot-assisted thoracic surgery (RATS). MITS interventions induce lesser inflammatory response and reduce the immune defense of the patient compared with open interventions (8), and the postoperative period is easier to manage in MITS, with less morbidity, and shorter postoperative drainage time and hospital stay (9, 10). With the popularization of MITS, mainly VATS, spontaneous ventilation (SV) was developed to further reduce the surgical stress in thoracic surgery (11). This supports the clinical observation that postoperative results are simpler and smoother in shorter and less invasive procedures (12). With the minimization of surgical stress in the thoracic surgical procedures, the procedure of administering anesthesia in thoracic operations had to be changed. Generally, in thoracic surgical procedures, the additional effect of mechanical one-lung ventilation (mOLV), with its positive and negative impact, on the perioperative inflammatory response must be evaluated. The negative inflammatory effect of mOLV can be reduced with spontaneous one-lung ventilation (sOLV) (13). One of the most relevant pathophysiological observations is the reduced inflammatory response and immune changes in patients on SV (14–16).

In this paper, the effects of different operative factors, such as surgical aggressiveness, additional local anesthesia, and type of ventilation, on postoperative SI and defense mechanism in lung cancer surgery are discussed.

## Pathophysiology of SI

In SI, the innate immune system and response are first activated. Primary immune cells (neutrophils, macrophages, natural killer cells, and dendritic cells) capable of phagocytosis and antigen presentation migrate to the surgically injured tissue. These primary immune cells recognize the damage-associated molecular patterns of the damaged tissue (e.g., surgical incision and preparation) via toll-like receptors and release proinflammatory cytokines, such as TNFα, IL-6, IL-8, and IL-1β, and anti-inflammatory cytokines, such as IL-4, IL-10, and IL-13. The release of the cytokines can be very quick. The level of IL-6, IL-8, and IL-10 may elevate at the time of skin closure after the lung resection, posing a risk of postoperative complications (17). Normal levels of these cytokines are necessary for physiological functions of the immune system. If inflammation can be controlled by the cellular defense and cytokines, it remains localized. SI can develop in advanced cases. The excessive production of proinflammatory cytokines has a negative effect on the normal functioning of the body, leading to the loss of organ function and possible multi-organ failure. The pathomechanism underlying the negative effect of cytokines in the advanced stages of SI can involve damage to the cell membrane, disseminated intravascular coagulation, and ischemia-reperfusion injury with capillary dysfunction, causing postoperative complications (18). A correlation between the levels of proinflammatory cytokines and the degree of postoperative SI has been proven (6, 19, 20).

During the SIRS, there is a massive release of immature neutrophils from the bone marrow into the circulation, causing an elevated leukocyte count after the surgery, and a lowering in the number of lymphocytes in the postoperative days by post-surgical apoptosis (19, 21). NK cells are activated by the high mobility group box protein 1, which is released from the damaged tissue. For specific immune responses, there must be an interaction between antigen-presenting cells (e.g., macrophages and dendritic cells) and T lymphocytes. The interaction of antigens and cytokines with CD4^+^ lymphocytes can result in their differentiation into Th1 (cellular immune response) or Th2 (humoral immune response) cells, both of which are regulated by cytokines (19, 20).

## Anti-Tumor Immunity and SI

A special concern—intraoperative tumor dissemination and control of circulatory tumor cells—is emerging in cases of cancer surgery (22). In an animal study, circulatory tumor cells were significantly elevated after tumor punction but disappeared after resection. Unfortunately, circulatory tumor cells could be detected in 6 weeks after resection. In view of this, the normal functioning of the immune system is very important in the postoperative 6 weeks to eliminate the circulatory tumor cells (23). If the number of lymphocytes and NK cells is reduced, their capacity against tumor cells is also decreased (24).

Clinical studies have proven the superiority of VATS over thoracotomy, showing favorable sequelae on cellular immune functions. VATS is associated with a lesser postoperative decrease in the numbers of circulating CD3^+^, CD4^+^, and CD8^+^ T cells, which lowers the risk of imbalanced immunoregulation and preserves immunosurveillance, decreasing the risk of tumor growth and recurrence (24, 25). Unlike open thoracotomy, intubated VATS major lung resection and lobectomy do not significantly reduce the T lymphocyte populations (8, 26). Additionally, non-intubated VATS unveiled new approaches for patients with non-small cell lung cancer (NSCLC), as the absence of intubation is associated with less reduction in NK cell and lymphocyte populations (15, 27). The reduction in lymphocyte number is not fully understood but has been widely investigated. It may be due to the redistribution of lymphocytes to the surgical site, apoptosis, regulation of T cells with reduced helper-inducer T cells, and increased cytotoxic T cells (28–30).

T lymphocytes are essential for immunoregulation and tumor suppression. An imbalance of CD8^+^ cytotoxic and CD4^+^ helper/regulatory T cells in the tumor-infiltrating lymphocyte (TIL) population in surgically treated NSCLCs was assessed to be a prognostic indicator after surgery (31). T lymphocytes mediate anti-tumor responses and learn to recognize tumor-specific antigens bound to the major histocompatibility complex (MHC)-I. After stimulation by antigen-presenting cells, CD8^+^ T cells are licensed to kill tumor cells and stimulate the production of a wide range of pro-inflammatory cytokines, resulting in appropriate immune-mediated destruction. However, tumor cells develop several mechanisms, such as expression of inhibitory receptors and cytokines, to evade the host immunity. Under chronic inflammation or tumor growth, T lymphocytes experience a persistently high antigen load, leading to the expression of inhibitory receptors on their surface, which limits the evolving activation of inflammatory cells. Programmed-death 1 (PD-1) inhibitory receptor, which is an immune checkpoint, and its ligand programmed death ligand 1 (PDL-1) are essential for immune tolerance as T lymphocytes can upregulate PD-1 receptor; thus, they lose the ability of progressive proliferation and activation (32, 33). However, tumor cells can express PDL-1 and promote binding to the PD-1 receptor; therefore, tumors co-opt this pathway to suppress effector T cell-mediated cell killing and avoid cell death (34). Recently, PDL-1 inhibitors have become the focus of treatment in several cancers, such as Hodgkin’s lymphoma, melanoma, and advanced NSCLC. IgG4 monoclonal antibodies that block PD-1 have been proven to be effective in patients with advanced NSCLC, and they increase the progression-free survival (PFS) and overall survival (OS), compared with conventional chemotherapies (35, 36). In a study on stage IV NSCLC immune checkpoint inhibitor (ICI) treatment, it was verified that elevated inflammatory cytokines, including IL-6, and the neutrophyl-lymphocyte rate before the immune therapy had a negative impact on the response, and the PFS was significantly shorter (37). The negative impact of SI on the success of ICIs has been proven in another study (38).

## Impact of the Surgical Approach on SI

Postoperative morbidity after VATS lobectomy has been proven to be significantly lower than that after open lobectomy. VATS is associated with reduced atrial fibrillation, renal failure, shorter hospital stay and chest tube drainage, and postoperative pneumonia (9, 10). Although these studies did not mention the reasons for the differences in postoperative pneumonia and the immune benefits of VATS/MITS, these were investigated later. The postoperative proinflammatory response is greater after open surgery than after VATS, which affects the innate immune response (39). IL-6 levels were significantly higher in open cases than in VATS (14), SIRS/SI has a significant correlation with elevated IL-6 levels (6), and the levels of IL-6 and IL-8 correlated with thoractomy, length of the surgery, and blood loss in esophageal surgery (7).

RATS, a type of MITS procedure, is increasingly being used in lung cancer surgery. The perioperative results of RATS are similar to those of the VATS, as noted in a meta-analysis (40), but the length of the surgery in some cases can be longer after RATS than after VATS. There were no differences in acute phase proteins and immune responses between the RATS and VATS procedures (41), showing that surgical trauma is similar in the two MITS methods.

Comparing the different types of MITS procedures, Tacconi reported that there was no difference in the SI between uniportal, multiportal, and hybrid VATS lobectomies. They reported that the level of SI markers returned to the preoperative levels after 5 days (42).

## Impact of SV on SI

During lung resections, mOLV is a highly recommended method for providing the technical background of preparations in the chest cavity. With this type of anesthesia, the dependent/ventilated lung is used instead of the two lungs for gas exchange, which can have negative effects. In mOLV, to maintain physiological oxygen and carbon dioxide levels, anesthesia uses a higher tidal volume and oxygen concentration, positive end-expiratory pressure (PEEP), or positive pressure ventilation. Despite the protective ventilation method (43), mainly in patients with underlying lung diseases (fibrosis, emphysema, and obstructive pulmonary disease) and pulmonary hypertension, ventilation can cause injury to the alveoli through overdistension of the alveoli, resulting in volutrauma/barotrauma and atelectrauma (44, 45). These changes are the source of the accumulation of inflammatory cells (neutrophils, macrophages, and lymphocytes), release of cytokines (TNF-α, IL-6, IL-8, and IL-1β, and edema in the dependent lung (biotrauma), causing SI (46, 47). The abovementioned side effects of mOLV can be reduced with sOLV.

In both non-intubated and intubated sOLV thoracic surgeries (48, 49), SV can prevent or reduce volutrauma, atelectrauma, and biotrauma of the mOLV. Better immune responses and less immunosuppression after SV surgery have been widely investigated and published. The changes in NK cells and lymphocytes during SV surgery were lower than those in relaxed surgery cases, and the return to the baseline level required lesser time (16). These studies revealed that there is less immunosuppression after SV surgery (15) and demonstrated that SV has a long-term impact on survival. In malignant pleural effusion cases, survival was longer in patients with SV than in those who underwent relaxed surgery. SV not only affects the immune cells but also the cytokine release. In a lung metastasectomy study, the impact of the non-intubated procedure was compared with that in relaxed surgery cases. Non-intubated procedure had less impact on immune function and SI, with less release of IL-6 (27). As a clinical consequence of the lower perioperative SI, the postoperative morbidity was lower, and the hospital stay was shorter after SV thoracic surgery. In a major lung resection study, the advantages of non-intubated, SV thoracic surgery on cytokine release were revealed. IL-6 and TNF-α levels were significantly lower in non-intubated patients than in relaxed surgery patients (11). The change in stress hormone levels as a factor in the SI pathway was moderate in SV surgery compared with that in relaxed surgical cases (50).

In addition to less volutrauma, atelectrauma, and biotrauma, SV reduces the changes in immune cells and cytokine release through another pathway. This involves a sympatholytic effect of locoregional anesthesia. Both, epidural and paravertebral/intercostal anesthesia with vagus blockade can reduce surgical stress, and the levels of IL-6, IL-8, and TNF-α (51, 52), and epidural anesthesia reduces cytokine levels in open esophagectomy cases, as well (53).

## Treatment of SI

Although many studies have reported the reduction of cytokine levels as a possible treatment for postoperative SI, prevention still remains the best treatment for it. Regarding the method of anesthesia administration, protective ventilation is important to prevent volutrauma (43), but the type of drugs used during narcosis can have a role in SI prevention (54). Although it is stated that sevoflurane can reduce the level of cytokines during mOLV (55), the beneficial effect of propofol over isoflurane is verified (56). Another approach to treat the pathophysiological manifestation of the SI could be to eliminate or at least reduce cytokine levels. There are many methods (filtration, dialysis [diffusion], adsorption) to overcome the challenge of reducing cytokine levels, but a real breakthrough has not been achieved yet (57). Currently, one of the most promising methods is the CytoSorb hemoadsorption, which has a positive effect in the advanced phase of the SI, like sepsis and pneumonia (58). However, the prevention of SI by eliminating cytokines in the early perioperative period or during the lung surgery has not been investigated yet, by our knowledge. In a cardiac surgery study of cardiopulmonary by-pass, there was no difference between the intraoperative and early postoperative cytokine levels and clinical results of the CytoSorb adsorption and control group (59).

## Discussion

The main issue in postoperative SI is how the pathological background of pro- and anti-inflammatory responses can affect the clinical picture. Patients who have undergone lung cancer surgery expect a quick recovery from the operation and long-term survival, and the surgical treatment should be adapted to this expectation. Because every surgical intervention causes an SI response, the best results can be achieved with less harmful surgery; in other words, the more minimally invasive is the thoracic surgery, the more preserved is the immune function. Based on the changes in the cellular defense and cytokine levels, this kind of postoperative SI period can take for around 3–12 days, but it affects not only on the early postoperative morbidity, but also the 30-day mortality (15, 20, 27).

Regarding the early postoperative morbidity, the reduced levels and functions of lymphocytes and NK cells can cause diminished cellular defense. In clinical practice, this can manifest as postoperative pneumonia, wound infection, or other inflammation. Postoperative pneumonia rates were 5% and 10%, and wound infection rates were 0.4% and 1% after VATS and open lobectomies, respectively (10).

Because of the less pro-inflammatory response, some postoperative morbidities can be reduced further with the use of SV (49). In SV surgery, postoperative morbidity is lower (5%) than in relaxed surgery cases (23%) (27). Generally, after SV VATS lung resection, the postoperative fasting time, drainage time, and hospital stay were shorter than those in relaxed VATS cases (60, 61).

The long-term oncological effects of SI are discussed based on different approaches for lung cancer resection. A meta-analysis showed the advantage of relaxed VATS in long-term survival after lung cancer resection, and the reason was suspected to be the lower levels of cytokines released after VATS compared with that in open cases (62). In another review article comparing long-term survival after VATS and open surgery, no significant difference in survival was found between the two approaches (63). The same uncertain advantage exists in the long-term survival of patients undergoing SV VATS procedures. Although immune function is less reduced after SV surgery, there are very few reports about the benefits of this approach in terms of long-term survival. One of the basic publications about the immune effect and impact on survival after SV showed better survival in malignant pleural fluid surgery (15), as well as significantly better survival and disease-free survival after SV for lung cancer resections compared with that in relaxed surgery cases (64). In contrast, there was no advantage in survival or recurrence was found after SV (65).

There are some encouraging results regarding the short-term oncological effect of SI. Better compliance with adjuvant chemotherapy was found after non-intubated VATS lobectomies than after relaxed VATS lobectomies, with less toxicity, and more patients (92%) could receive the adjuvant chemotherapy protocol, compared with 72% in relaxed surgery cases (66). Less toxic and more adjuvant treatment should provide better oncological outcomes. The same thought process can be seen in the case of ICI treatment, although in the current studies on the application of ICIs in non-operated patients, the results and the message are clear: more SI before the beginning of immunotherapy is associated with less successful outcomes. Surgery for lung cancer should provide the lowest postoperative SI before the planned ICIs. Lymphocytes are crucial in anti-tumor response; thus, the impact of intubated and non-intubated VATS on the prognosis of advanced NSCLC patients receiving immunotherapies remains to be elucidated (37, 38).

In conclusion, to reduce postoperative SI, thoracic surgeons should reduce the damage caused by the surgical procedures for lung cancer while adhering to the oncological principles. The more minimally invasive the procedures used, the less immunosuppression is required by the patient. The positive effect of VATS on SI (less diminished lymphocyte function, less pro-inflammatory cytokine release) is mirrored in the better postoperative results, and to continue this beneficial minimalization of surgical aggressivity, SV surgery could be an option. Although SV is not a widely accepted procedure, it has a very simple and useful message: the positive effect of locoregional anesthesia on SI, which can be used properly in relaxed VATS and open cases.

## Author Contributions

JF: writing the paper, collecting data, and conception of the manuscript. TN: writing the paper, collecting data. JL: writing the paper and collecting data, CSF: conception of the anesthesiologic aspect of the topic TG: collecting data, NZT: collecting data, DP: conception of the immunological aspect of the topic, ZSZ conception and finalizing of the manuscript. ZSSZ: conception of the anesthesiologic aspect of the topic. All authors contributed to the article and approved the submitted version.
